# Exploring the Chemical Space of Protein Glycosylation in Noncovalent Protein Complexes: An Expedition along Different Structural Levels of Human Chorionic Gonadotropin by Employing Mass Spectrometry

**DOI:** 10.1021/acs.analchem.1c02199

**Published:** 2021-07-21

**Authors:** Maximilian Lebede, Fiammetta Di Marco, Wolfgang Esser-Skala, René Hennig, Therese Wohlschlager, Christian G. Huber

**Affiliations:** † Department of Biosciences, Bioanalytical Research Labs, University of Salzburg, Hellbrunner Straße 34, 5020 Salzburg, Austria; ‡ Christian Doppler Laboratory for Innovative Tools for Biosimilar Characterization, University of Salzburg, Hellbrunner Straße 34, 5020 Salzburg, Austria; § Department of Biosciences, Computational Systems Biology Group, University of Salzburg, Hellbrunner Straße 34, 5020 Salzburg, Austria; ∥ glyXera GmbH, Brenneckestraße 20 - ZENIT, 39120 Magdeburg, Germany; ⊥ Max Planck Institute for Dynamics of Complex Technical Systems, Sandtorstraße 1, 39106 Magdeburg, Germany

## Abstract

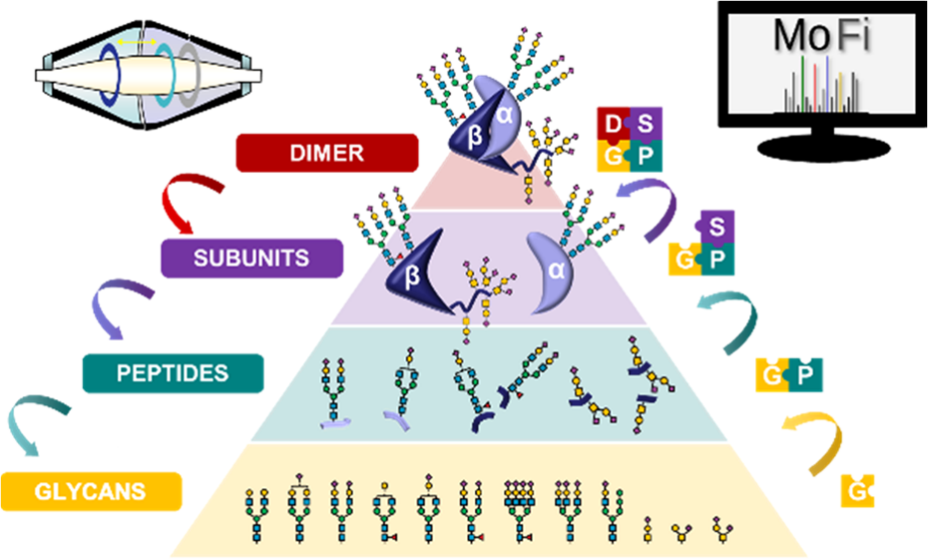

Modern analytical approaches employing high-resolution mass spectrometry (MS) facilitate the generation of a vast amount of structural data of highly complex glycoproteins. Nevertheless, systematic interpretation of this data at different structural levels remains an analytical challenge. The glycoprotein utilized as a model system in this study, human chorionic gonadotropin (hCG), exists as a heterodimer composed of two heavily glycosylated subunits. In order to unravel the multitude of glycoforms of recombinant hCG (drug product Ovitrelle), we combine established techniques, such as released glycan and glycopeptide analysis, with novel approaches employing high-performance liquid chromatography-mass spectrometry (HPLC-MS) to characterize protein subunits and native MS to analyze the noncovalent hCG complex. Starting from the deconvoluted mass spectrum of dimeric hCG comprising about 50 signals, it was possible to explore the chemical space of hCG glycoforms and elucidate the complexity that hides behind just 50 signals. Systematic, stepwise integration of data obtained at the levels of released glycans, glycopeptides, and subunits using a computational annotation tool allowed us to reveal 1031 underlying glycoforms. Additionally, critical quality attributes such as sialylation and core fucosylation were compared for two batches of Ovitrelle to assess the potential product variability.

## Introduction

The existence of proteoforms—protein variants due to sequence variation and/or post-translational modifications (PTMs)—significantly augments the chemical space of protein structures and has been recognized as a major contribution to the functional diversity and regulation of proteins.^1^ Thus, the structural and functional characterization of proteoforms is a prerequisite for understanding how chemical structures are involved in the complex network of biochemical reactions responsible for the functioning of living organisms. Among the multitude of PTMs, glycosylation is the one contributing most to protein heterogeneity.

Glycosylation, i.e. the conjugation of carbohydrates with a protein backbone, is crucial to the structure and function of certain proteins. A plethora of coexisting glycoforms arises from macroheterogeneity, the presence or absence of a carbohydrate structure at a specific glycosylation site within the protein, as well as microheterogeneity, the occurrence of different glycan structures at a specific site.^2^ Due to the complexity of glycan structures and the subtle structural differences between glycoforms, characterization of glycoproteins poses a substantial analytical challenge.

To address this challenge, analytical techniques such as high-performance liquid chromatography (HPLC) and mass spectrometry (MS) have been employed at different levels of structural complexity, ranging from released glycans, glycopeptides, to intact proteins, and protein complexes.^3,4^ Released glycan analysis upon removal of the glycans from the protein backbone provides information on glycan structure but not on the localization within the protein.^5^ In contrast, glycopeptide analysis allows acquisition of site-specific glycosylation data.^6^ The molecular context can be preserved by employing MS at the intact protein level to acquire information on the whole glycoprotein and to reveal the co-occurrence of different glycoforms.^7^ Finally, to unravel the biomacromolecular higher-order structure, noncovalent protein complexes can be analyzed by MS under nondenaturing conditions, referred to as native MS.^8−13^ In the context of glycoproteins, native MS permits elucidation of coexisting multiglycoform complexes arising from combinations of different glycoforms.^14,15^

Nevertheless, isobaric carbohydrate building units as well as the high complexity of glycan structures lead to the presence of many glycoforms with identical mass, thus indistinguishable by MS. Furthermore, native measurement of protein complexes is often limited in resolving glycoforms of similar mass. Specifically, the natural width of isotopologue clusters hampers the resolution of small mass differences in the mass spectra of proteins or protein complexes.^16,17^ At this level, one cannot discriminate between glycoforms comprising two fucose residues (292 Da) and those having one sialic acid residue (291 Da). Hence, enzymatic dissection may be employed to reduce spectral complexity and to enable unambiguous identification of different glycoforms.^18^

As no individual analytical technique suffices to comprehensively characterize protein glycosylation, current developments tend toward a combination of approaches at different structural levels to assess glycosylation. This so-called hybrid MS strategy overcomes the shortcoming of the individual methods but generates a large amount of interdependent data, necessitating bioinformatic tools for evaluation.^19−23^

In this study, we assess different levels of glycoprotein characterization, ranging from released glycans to intact noncovalent glycoprotein complexes. As a model for proteoform heterogeneity, we utilize human chorionic gonadotropin (hCG), a highly glycosylated protein hormone involved in early embryo-maternal communication and promotion of pregnancy.^24−26^ The protein exists as a heterodimer comprising two noncovalently interacting subunits, α (hCGα) and β (hCGβ). N-glycosylation occurs at two sites in each of hCGα (N52, N78) and hCGβ (N13, N30).^27^ O-glycosylation sites are unique to hCGβ and have been described in its unfolded serine-rich C-terminal region (S121, S127, S132, S138).^28^ A fifth O-glycosylation site (S130 or T140) has been proposed by Bai et al. based on an approach combining enzymatic dissection and tandem MS (MS/MS).^29^

As a therapeutic, hCG is applied in the treatment of infertility in men and women.^30^ One of the commercially available drug products, Ovitrelle, contains recombinant hCG, expressed in Chinese hamster ovary (CHO) cells. From a functional perspective, O-glycosylation and sialylation in gonadotropins increase serum half-life and biological activity, while removal of N-glycans reduces receptor binding affinity.^31^ Glycosylation therefore constitutes a critical quality attribute (CQA) of gonadotropin drug products.

Characterization of hCG has been addressed by HPLC or capillary electrophoresis (CE), mostly coupled with MS, at the level of released glycans,^32^ glycopeptides,^28,29,33,34^ and, rarely, at intact protein level.^35−40^ Camperi et al.^36−38,40,41^ employed different techniques for separation of hCGα glycoforms; annotation of mass spectra was attempted by a noncombinatorial approach, resulting in only approximately forty identified glycoforms.^41^ With regard to hCGβ, neither separation of glycoforms nor acquisition of mass spectra of this subunit was achieved. Toll et al.^35^ previously accomplished analysis of both intact hCGα and hCGβ by HPLC-MS. Applying a combinatorial approach, they were able to assign specific glycoforms for hCGα. However, the high spectral complexity and isobaricity of variants prevented unambiguous peak assignment for hCGβ.

Here, we introduce a global approach to characterize the glycosylation patterns of recombinant hCG in the drug product Ovitrelle at multiple structural levels by applying multiplexed capillary gel electrophoresis with laser-induced fluorescence detection (xCGE-LIF), HPLC-MS, native MS, and the computational annotation tool MoFi.^18,42^ In order to integrate the data, we build libraries at the level of released glycans, glycopeptides, and subunits for stepwise knowledge transfer across all structural levels up to the noncovalent protein complex. Furthermore, we scrutinize the underlying glycoforms by enzymatic dissection of intact protein subunits as well as of the noncovalent complex to reduce spectral complexity. For the first time, we acquire isotopically resolved spectra of the intact hCGβ subunit as well as native mass spectra of the hCG dimer, which proves useful for highly informative batch-to-batch comparisons of biopharmaceuticals.

## Experimental Section

### Materials

Different batches of Ovitrelle (Roche. Lot: BA059433, BA056714, expiration date 04/2021 and 01/2021, respectively) were purchased from a local pharmacy. Dithiothreitol (DTT), guanidine hydrochloride (Gnd-HCl), ammonium acetate (AmAc), ammonium bicarbonate, iodoacetamide (IAA), and formic acid (FA) were purchased from Sigma-Aldrich (St. Louis, MO). Sodium acetate was purchased from Fluka Analytical (Steinheim, Germany). Trypsin was purchased from Promega (Fitchburg, WI). Sialidase (Neuraminidase from *Arthrobacter ureafaciens*) was purchased from Roche Applied Science (Mannheim, Germany) and PNGase F from New England Biolabs. LC-MS grade acetonitrile (ACN) was purchased from VWR chemicals (Radnor, PA). Water (H_2_O) was purified in-house by a MilliQ Integral 3 system from Merck Millipore (Burlington, MA).

### Sample Preparation and Enzymatic Treatment

Released N-glycans were prepared, according to Huffman et al.,^43^ using a glyXprep kit (KIT-glyX-NGly.PP-APTS-48-01, glyXera, Magdeburg, Germany), based on multiplexed capillary electrophoresis with laser-induced fluorescence detection (xCGE-LIF). Following the kit instruction guide, 10 μg of recombinant human chorionic gonadotropin dissolved in 6 μL of ultrapure water was supplemented with 1 μL of Denaturation Solution (kit) and 1 μL of 1 mol L^-1^ DTT. For protein denaturation and linearization, the mixture was incubated for 10 min at 60 °C. Additives were neutralized by adding 2 μL of Neutralization Solution (kit). Glycans were released by adding 1 μL of PNGase F Solution (kit) and incubating for 30 min at 37 °C. After glycan release, the samples were dried in a vacuum concentrator and labeled with the fluorescence dye 8-aminopyrene-1,3,6-trisulfonic acid (APTS). Therefore, samples were resolved in 2 μL of ultrapure water, 2 μL of APTS Labeling Solution (kit), and 2 μL of ReduX Solution (kit), mixed carefully, and incubated for 3 h at 37 °C. Labeling reaction was stopped by adding 100 μL of Stopping Solution (kit). According to the kit instruction guide, based on Hennig et al.,^44^ post-derivatization cleanup was performed by HILIC-SPE as follows: the samples were applied to a filter plate well containing 200 μL of glyXbead Slurry (kit) and incubated for 5 min at ambient temperature for binding, followed by washing and elution steps. To analyze the released O-glycans, 5 μg of recombinant human chorionic gonadotropin was dot-blotted onto a 0.2 μm pore size poly(vinylidene difluoride) (PVDF) membrane (Millipore, Darmstadt, Germany) and glycans were released as described in detail by Jensen et al.^45^

For protein alkylation, 10 μL of drug product (5 μg of protein) was diluted with 50 mmol L^–1^ ammonium bicarbonate to a volume of 50 μL. The protein was denatured and reduced in a final volume of 110 μL with 40 mmol L^–1^ DTT in 3 mol L^-1^ Gnd-HCl for 1 h at 50 °C while shaking (900 rpm). Alkylation was done in 30 mmol L^–1^ IAA solution for 1 h at 22 °C in the dark while shaking (900 rpm). Buffer exchange was carried out using Amicon Ultra 0.5 mL centrifugal filters (Merck), with a molecular weight cutoff of 3 kDa and 50 mmol L^–1^ ammonium bicarbonate to a final volume of 50 μL.

For glycopeptide analysis, 3.5 μg of alkylated protein (35 μL) was digested with 1 μg of trypsin overnight at 37 °C while shaking. The sample was diluted 1:5 with 50 mmol L^–1^ ammonium bicarbonate to a final concentration of approximately 20 ng μL^–1^.

In order to remove N-glycans and sialic acids for assessment of O-glycosylation, 1.5 μg of alkylated protein was digested with 1.5 mU of sialidase and 250 U of PNGase F in 50 mmol L^–1^ ammonium bicarbonate in a final volume of 12 μL at 37 °C overnight.

Desialylation was performed using sialidase. Seventy-five microliters of the drug product Ovitrelle containing 32.5 μg of hCG was buffer exchanged using a Micro Bio-Spin P-6 column (Bio-Rad Laboratories) in 40 mmol L^–1^ sodium acetate at pH 5.0. The protein was digested with 3.25 mU of sialidase to achieve an enzyme-to-hCG ratio of 0.1 mU μg^–1^. The reaction was carried out overnight at 37 °C while shaking. The sample was then buffer exchanged in 150 mmol L^–1^ AmAc twice using Micro Bio-Spin P-6 columns.

For native analyses, hCG in the drug product Ovitrelle was buffer exchanged twice using a Micro Bio-Spin P-6 column in 150 mmol L^–1^ AmAc.

### xCGE-LIF-Based Analysis of Released N-Glycans

Analyses of released N-glycans were conducted on a glyXboxCE system (glyXera, Magdeburg, Germany), based on xCGE-LIF. For migration time alignment, crucial for glycan peak annotation via migration time matching with the database entries of glyXbaseCE (glyXera, Magdeburg, Germany), 1 μL of sample was mixed with 1 μL of 2nd NormMiX (STD-glyX-2ndN-APTS-100Rn-01, glyXera, Magdeburg, Germany) and 1 μL of prediluted GeneScan 500 LIZ Size Standard (4322682, Thermo Fisher Scientific).^46^ The mixture was combined with 6 μL of glyXinject (C-glyXinj-1.6mL-01, glyXera, Magdeburg, Germany) and subjected to xCGE-LIF analysis. The xCGE-LIF measurements were performed on a glyXboxCE system (based on a modified 16 capillary 3130xl Genetic Analyzer), equipped with a 50 cm capillary array and filled with a POP-7 polymer (4363929, Thermo Fisher Scientific). The samples were electrokinetically injected and analyzed with a running voltage of 15 kV for 40 min. The generated glycan data were analyzed with the glycoanalysis software glyXtoolCE (glyXera, Magdeburg, Germany), performing migration time alignment, raw data smoothing, peak picking, relative quantification, and peak annotation. Glycan peak annotations were additionally confirmed by repeated measurement of the samples after exoglycosidase digestions using α(2-3,6,8) sialidase (GK80040, Agilent) and α(1-2,4,6) fucosidase (P0749L, New England BioLabs), following the enzyme supplier’s instructions.

### Porous Graphitized Carbon Liquid Chromatography Electrospray Ionization Mass Spectrometry (PGC-LC-MS)-Based Analysis of Released O-Glycans

Released and desalted O-glycans of each sample were dissolved in 10 μL of MilliQ-water and 3 μL was injected for each porous graphitized carbon liquid chromatography electrospray ionization mass spectrometry (nano-PGC-LC-MS) run. The employed nanoLC setup was as follows: a PGC precolumn (HYPERCARB 5 μm, 30 mm × 0.32 mm, Thermo Fisher Scientific, Waltham, MA) and a PGC separation column (HYPERCARB 3 μm, 100 mm × 0.075 mm, Thermo Fisher Scientific) were installed in an Ultimate 3000 UHPLC system (Dionex, Germering, Germany). The nanoLC setup was directly coupled to an amaZon speed ETD ion trap mass spectrometer equipped with a CaptiveSpray source (Bruker, Bremen, Germany) for online detection of glycans in negative ion mode. O-glycan nanoLC separation as well as MS spectra acquisition, interpretation, and annotation were conducted as previously described.^45^

### RP-HPLC-MS/MS Analysis of Tryptic Glycopeptides

Glycopeptide measurements were carried out on an Ultimate 3000 RSLCnano UHPLC (Thermo Fisher Scientific) coupled with a Q Exactive Plus Hybrid Quadrupole-Orbitrap mass spectrometer (Thermo Fisher Scientific). The injection volume was 1.0 μL. Chromatographic separation was achieved using a 75 μm × 500 mm Acclaim Pepmap 100 C18 LC column (Thermo Fisher Scientific) operated at 50 °C and a flow rate of 300 nL min^–1^. Eluent A comprised water with 0.1% FA and eluent B comprised acetonitrile with 0.1% FA. A gradient from 1 to 30% B over 75 min was applied, followed by an increase from 30 to 60% B in 15 min. 99% B was held for 10 min and equilibration was carried out at 1% B for 35 min. The ion-source spray voltage was set to 1.5 kV, capillary temperature to 250 °C, in-source CID to 0, and S-lens RF level to 60. For MS1, the Orbitrap mass analyzer *m*/*z* range was set to *m*/*z* 400–2000 with a resolution setting of 70 000 at *m*/*z* 200 and microscan 1, and the AGC target was 3 × 10^6^ with a maximum IT of 100 ms. For MS/MS, the mass range was *m*/*z* 200–2000 with a resolution setting of 17 500 at *m*/*z* 200. The AGC target value was 1 × 10^5^. The maximum injection time was set to 50 ms. The normalized collision energy (NCE) was 28. Microscans were not averaged.

### RP-HPLC-MS Analysis of Intact hCG Subunits

Intact protein analysis was performed on an Ultimate 3000 UHPLC system (Thermo Fisher Scientific, Germering, Germany) combined with a Thermo Scientific Q Exactive mass spectrometer (Thermo Fisher Scientific, Bremen, Germany), operated with Chromeleon 7.2.6. Chromatographic separation was achieved using a 2.1 mm × 150 mm Discovery Wide Pore C18 column (Supelco, Bellefonte, PA) with a particle diameter of 3 μm. The injection volume was 5.0 μL. Gradient elution from 15 to 30% mobile phase B was carried out over a time span of 67.5 min at a flow rate of 100 μL min^–1^ and a temperature of 60 °C. Mobile phase A comprised water and 0.10% FA; mobile phase B comprised acetonitrile and 0.10% FA. The ion-source spray voltage was set to 4.5 kV. The Orbitrap mass analyzer mass range was set to 1300–3000 *m*/*z* with a resolution of 140 000 at 200 *m*/*z*. The S-lens RF level was set to 50 and the automatic gain control (AGC) target value was 3 × 10^6^ with a maximum injection time (IT) of 150 ms. To improve the signal-to-noise ratio, four microscans were averaged. Enzymatically dissected hCG was analyzed using the same parameters.

### Native MS Analysis of the hCG Dimer

Native MS analyses were carried out at an hCG concentration of approximately 0.5 μg μL^–1^ in 150 mmol L^–1^ AmAc. The analyses were performed with a Q Exactive Plus Hybrid Quadrupole-Orbitrap mass spectrometer equipped with a Thermo Nanospray source with a Static NSI Probe (Thermo Fisher Scientific, Bremen, Germany). Twenty microliters of the sample was loaded in the extra coated borosilicate emitter for static nanospray (cat No. ES387, Thermo Scientific, San Jose, CA) using a gel-loading pipette tip and directly infused in the MS. The Q Exactive Plus was used with the BioPharma platform in Protein Mode, which allows for ions to be transferred, trapped, and cooled in the HCD cell while applying a reduced trap and HCD gas pressure, resulting in increased signal intensities. The source and MS parameters were set as follows: spray voltage 2 kV, capillary temperature 250 °C, in-source CID from 30 to 60, S-lens RF level from 100 to 200, polarity positive, trapping gas pressure setting 1, and averaging 1000. Different resolution settings were applied in order to obtain isotopically resolved spectra. Since increasing the resolution only led to an increase in the noise level but not to isotopically resolved spectra, the lowest resolution setting was chosen (17 500 at *m*/*z* 200). The *m*/*z* range was chosen depending on the sample at 2000–5000, 1500–6000, or 500–5000. Spectra were averaged from 24 to 118 scans (at microscans 10 each) during acquisition with the averaging parameter set at 1000 in order to increase the signal-to-noise ratio. Ovitrelle batches were compared using identical parameter settings. Base calibration was done using LTQ Velos ESI positive ion calibration solution, while ammonium hexafluorophosphate was used for calibration of the high mass range, in order to cover the *m*/*z* range from 195.09 to 5559.98.

### MS Data Processing and Peak Assignment

Glycopeptide data was evaluated using Byonic v3.10.10, Byologic v3.10-52-g5de54ba3ecx64, and Byos v3.10-52-g5de54ba3ecx64 (Protein Metrics Inc., Cupertino, CA). A glycan library was built by integration of released glycan data and used for glycopeptide identification/quantification. Digestion specificity was set to “fully specific” with a maximum missed cleavage of 1. Precursor mass tolerance and CID/HCD mass tolerance were set to 10 and 20 ppm, respectively. Carbamidomethylation/+57.021464 was set as a fixed modification and oxidation/+15.994915 as common 1. No automatic score cutoff was used. N- and O-glycans were searched separately. For quantification, also glycopeptides with missed cleavages were considered, as long as they did not carry another glycosylation site.

For subunit data, isotopically resolved raw spectra were deconvoluted using the Xtract algorithm embedded in Xcalibur 2.2 (Thermo Fisher Scientific).

For dimer data, deconvolution of raw to zero-charge spectra was accomplished using the ReSpect algorithm of BioPharma Finder 3.0 (Thermo Fisher Scientific). The default native method was selected and the parameters depending on the specific spectrum (*m*/*z* range, selected retention time, model mass range, charge state range, minimum adjacent charge) were changed. Only the three most abundant charge states (11+ to 13+) were used to deconvolute the intact dimer.

Peak assignment of deconvoluted mass spectra was performed using the assignment tool MoFi, which assigns monosaccharide composition for each peak by application of a two-stage search algorithm that finds possible glycan combinations and compiles a hierarchical list of glycoforms. From glycopeptide data, a site-specific quantitative glycopeptide library was generated and used to assign peaks in deconvoluted spectra of hCG subunits in MoFi with a mass tolerance of 20 ppm. To annotate dimer spectra, a library of intact hCGα and hCGβ was built and possible combinations of subunit glycoforms within dimer context were assessed using MoFi.^47^

The resulting glycoforms were ranked by probability of occurrence. A mass tolerance of ±3 Da was used between the theoretical and experimental mass.

For isotopically resolved spectra of hCG subunits, monoisotopic masses of monosaccharides and other PTMs and atomic weight of elements were used in MoFi for annotation, while for dimer spectra, averaged masses as defined by IUPAC in 2013 were used.

### Code and Data Availability

Raw files and MoFi settings are available from Zenodo. (DOI: 10.5281/zenodo.4320966). All input files and data analysis scripts used in this study are freely available from GitHub (https://github.com/cdl-biosimilars/hcg-glycosylation).

### Safety Statement

No unexpected or unusually high safety hazards were encountered.

## Results and Discussion

### Glycoform Profiles Represent “Fingerprints” of hCG Drug Products

**Figure 1:**
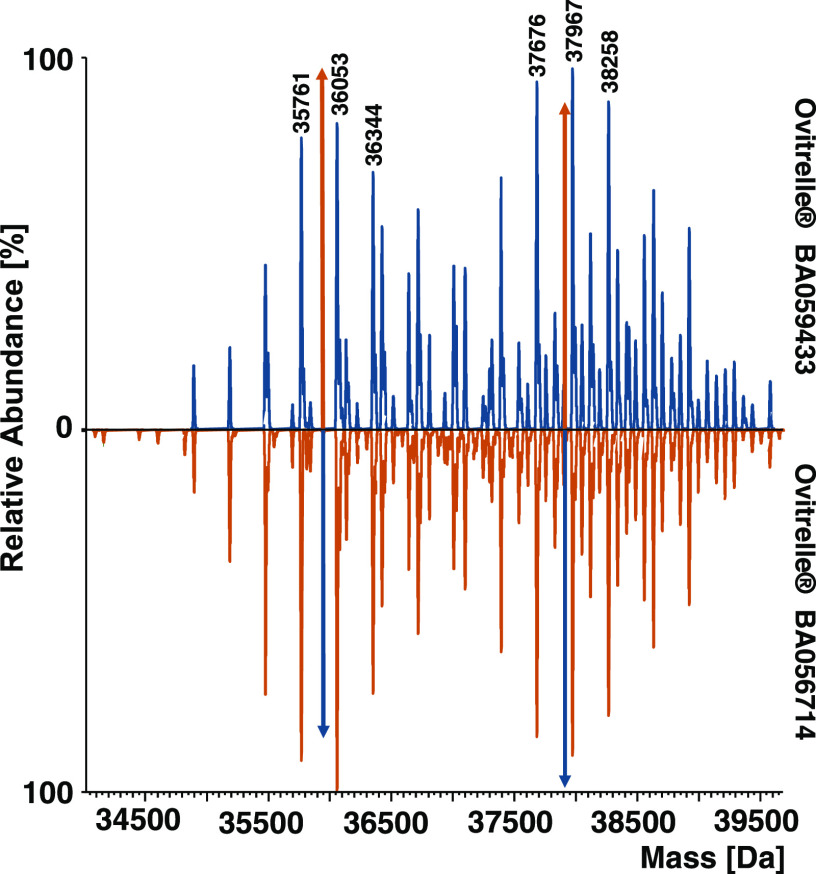
Mirror plot of deconvoluted mass spectra of dimeric hCG of Ovitrelle batches BA059433 and BA056714. Spectra were obtained with an instrument resolution setting of R = 17 500 at *m*/*z* 200. Colored arrows represent the peak height of the highest peak in the signal series of the mirrored batch.

Native MS is a powerful tool to obtain global information about the composition and PTMs of noncovalent protein complexes by avoiding lengthy sample preparation or potential introduction of artificial modifications. We analyzed two batches of the hCG-based drug product Ovitrelle to generate mass fingerprints for batch-to-batch comparison (for raw spectra, see Figure S1). To maintain the dimeric hCG complex in a quasi-native conformation, samples were buffer exchanged into 150 mM ammonium acetate and analyzed by direct infusion using static nano-ESI-MS. The resulting deconvoluted mass spectra of two Ovitrelle batches were highly complex with two distinct sets of peaks, ranging from 34.5 to 39.6 kDa (Figure 1). We concluded that the detected masses correspond to the hCG heterodimer existing in a multitude of glycoforms. Considering the difference between the theoretical mass of the protein backbone of the subunits (25.7 kDa) and observed masses, we attributed ≈30% of the molecular mass (12 kDa) to the attached carbohydrate structures. The spectra revealed reoccurring mass differences arising from either increasing content of sialic acids (291 Da) or multiple fucoses (2 × 146 = 292 Da, Figure 1). Moreover, mass differences of 365 and 146 Da were observed, corresponding to HexNAc_1_Hex_1_ and one fucose residue, respectively. Both batches of Ovitrelle resulted in signals with equal masses, which varied slightly in abundance, reflecting overall comparable glycoform patterns.

The mass spectra of the hCG complex obtained by native MS thus provided a holistic picture of heterogeneity arising from glycosylation. Furthermore, information on total glycan mass could be obtained. However, no information on individual glycan structures or site occupancy was gained, preventing the assignment of specific glycoforms to observed signals. Thus, we devised a systematic workflow for in-depth analysis of hCG glycoforms at different structural levels to gain further insight into the chemical space of hCG glycosylation, applicable in the comparison of two Ovitrelle batches (Figure S2).

### Released Glycan Analysis Provides Insight into the Origin of Complexity

Initially, N-glycans were released by treatment with PNGase F, labeled with aminopyrene-1,3,6-trisulfonic acid, and analyzed using xCGE-LIF (Figure S3). Forty-three peaks were detected and identified via migration time matching with database entries, and additionally confirmed with repeated measurements after exoglycosidase digests. Mainly, complex type N-glycans carrying *N*-acetylneuraminic acid (Neu5Ac) were observed. Specifically, biantennary N-glycans, such as A2S1G1 and A2S2, predominated (a summary of all possible glycan structures with their names and composition can be found in the Supporting Information, Released glycan data). Additionally, mono- and triantennary structures were detected at lower abundances. Some structures also exhibited core fucosylation.

Furthermore, we analyzed chemically released O-glycans by PGC-HPLC-MS/MS (Figure S4). O-glycan structures were assigned based on elution times and fragmentation patterns. The identified O-glycans were of the core-1 type (HexNAc_1_Hex_1_) with either one or two attached sialic acids. The varying degrees of sialylation and fucosylation of the identified N- and O-glycan structures are well in accordance with the delta mass series of 146 and 291 Da previously observed at the level of the hCG dimer (Figure 1).

### Glycopeptide Analysis Reveals the Site-Specific Context of Glycan Structures

All possible N- and O-glycan structures were combined in a glycan library (Supporting Information, Released glycan data) for the use in identification of glycopeptides. Glycopeptides were analyzed after tryptic digestion by nano-HPLC-MS/MS and quantified in a label-free approach by means of extracted ion current chromatograms (Figure S5). In accordance with the released glycan data, predominantly biantennary glycans were identified for the two N-glycosylation sites of both subunits at peptide level. However, we observed site-specific differences with regard to N-glycan structures, e.g. type, fucosylation, or sialylation (Figure S6).

Unambiguous site-specific assignment of O-glycans was hampered by the coexistence of eight serine residues and one threonine residue in close sequential proximity, which led to the generation of hCGβ peptides with multiple possible O-glycosylation sites. The detected tryptic glycopeptides (Table 1) carried up to two O-glycans each, indicating a maximum of six O-glycans present at the intact level (Figure S7). Therefore, we were unable to assign O-glycosylation to specific sites within those peptides.

**Table 1:** List of Detected Tryptic Glycopeptides and Possible Glycosylation Sites^a^

subunit	position	sequence	glycan type	site
hCGα	52–63	**N**VTSESTCCVAK	N	N52
hCGα	76–91	VE**N**HTACHCSTCYYHK	N	N78
hCGβ	9–20	CRPI**N**ATLAVEK	N	N13
hCGβ	21–43	EGCPVCITV**N**TTICAGYCPTMTR	N	N30
hCGβ	115–122	FQD**SSSS**K	O	S117–S120
hCGβ	123–133	APPP**S**LP**S**P**S**R	O	S127, S130, S132
hCGβ	132–145	LPGP**S**D**T**PILPQ	O	S138, T140

a N and O glycosylation sites are indicated by boldface letters.

### Analysis of Subunits Provides Information about the Molecular Context of Glycosylation

Data on N- and O-glycan structures and glycosylation site occupancy together with information about relative glycan abundances (site-specific glycan library) constitute the basis for the assignment of possible glycoforms at the level of hCG subunits.

Interpretation of the glycoform patterns at a mass tolerance of 20 ppm was realized by a two-step algorithm (modification finder, MoFi). MoFi combines monosaccharide composition determined via intact molecular mass measurements and the site-specific glycan library, as described in detail in ref (42). MoFi is able to calculate the contribution to peak height of individual isobaric glycoforms (hit scores) based on the site-specific relative abundance of glycans retrieved from glycopeptide analysis.

In order to obtain subunit spectra, RP-HPLC-MS analysis of Ovitrelle was carried out under denaturing conditions and revealed two sets of chromatographic peaks. The first can be attributed to hCGα and the second arises from hCGβ, according to molecular masses (see Figure S8 for the chromatogram and Figure S9d for raw spectra). Employing a mass spectrometric resolution setting of 140 000, we were able to record isotopically resolved spectra of both subunits (Figures S9d and S10).

Due to the high spectral complexity of subunits arising from many underlying glycoforms, reduction of molecular diversity upon glycosidase digestion was used to support the annotation of glycoform profiles, as outlined in detail in ref (18).

**Figure 2:**
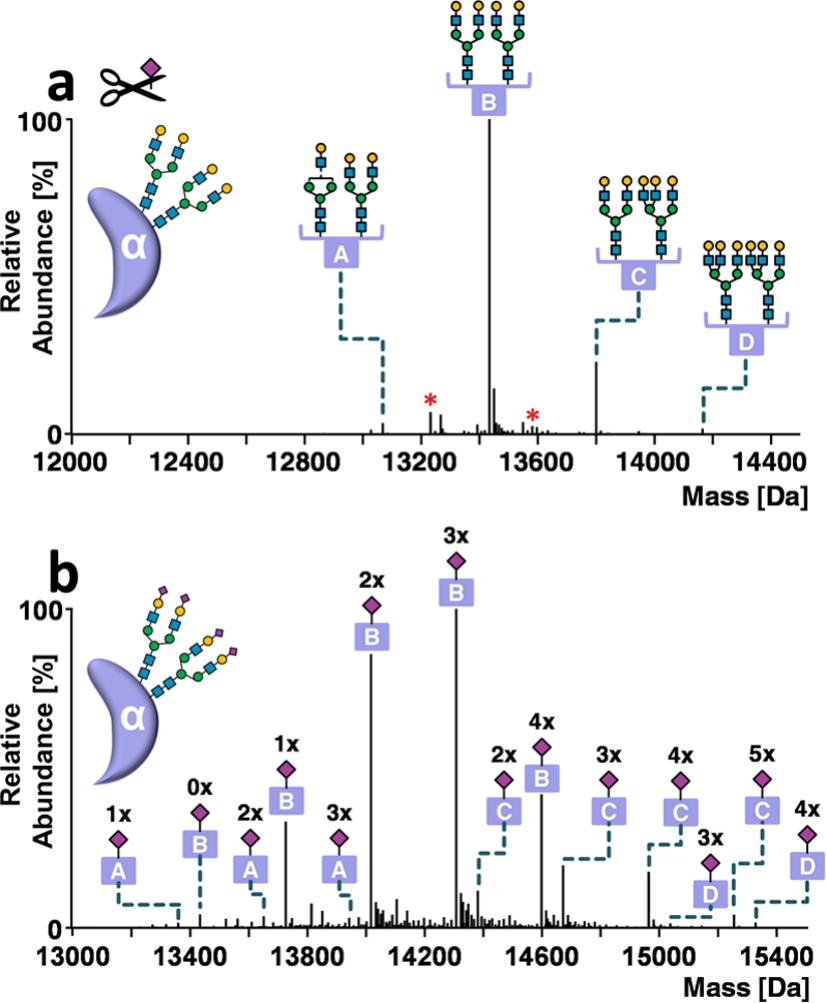
Annotation of hCGα glycoforms. Deconvoluted mass spectra of hCGα obtained by HPLC-MS analysis (a) after desialylation or (b) without enzymatic treatment. Glycoforms are indicated by letters (A–D). Red asterisks indicate fucose variants. Numbers above the peaks refer to Neu5Ac residues. Peak lists with all possible glycoform assignments for untreated and desialylated hCGα are available in Supporting Information, Annotations hCGα BA059433. The instrument resolution setting was 140 000 at *m*/*z* 200.

For desialylated hCGα, fucosylated and non-fucosylated bi- and triantennary N-glycan structures were readily assignable in the deconvoluted spectra of hCGα treated with sialidase (Figure 2a) using a site-specific glycan library, where sialic acids were removed in silico and abundances were recalculated. The 194 signals ranging from 13.0 to 15.5 kDa of untreated hCGα were consecutively annotated using the site-specific glycan library (Figure 2b). The most prominent glycoform series comprised two biantennary N-glycans differing in the number of terminal sialic acids, ranging from zero (A2G2/A2G2) to four (A2S2/A2S2) (letter B, Figure 2b).

Due to the existence of isobaric variants, multiple glycoforms were assigned for each mass, taking into account individual glycan contributions. For example, the most abundant hCGα signal at 14 307.1 Da was assigned to the glycoform A2S1G1/A2S2, contributing to 98.9% of peak abundance. The remaining 1.1% is explained by minor N-glycoforms.

**Figure 3:**
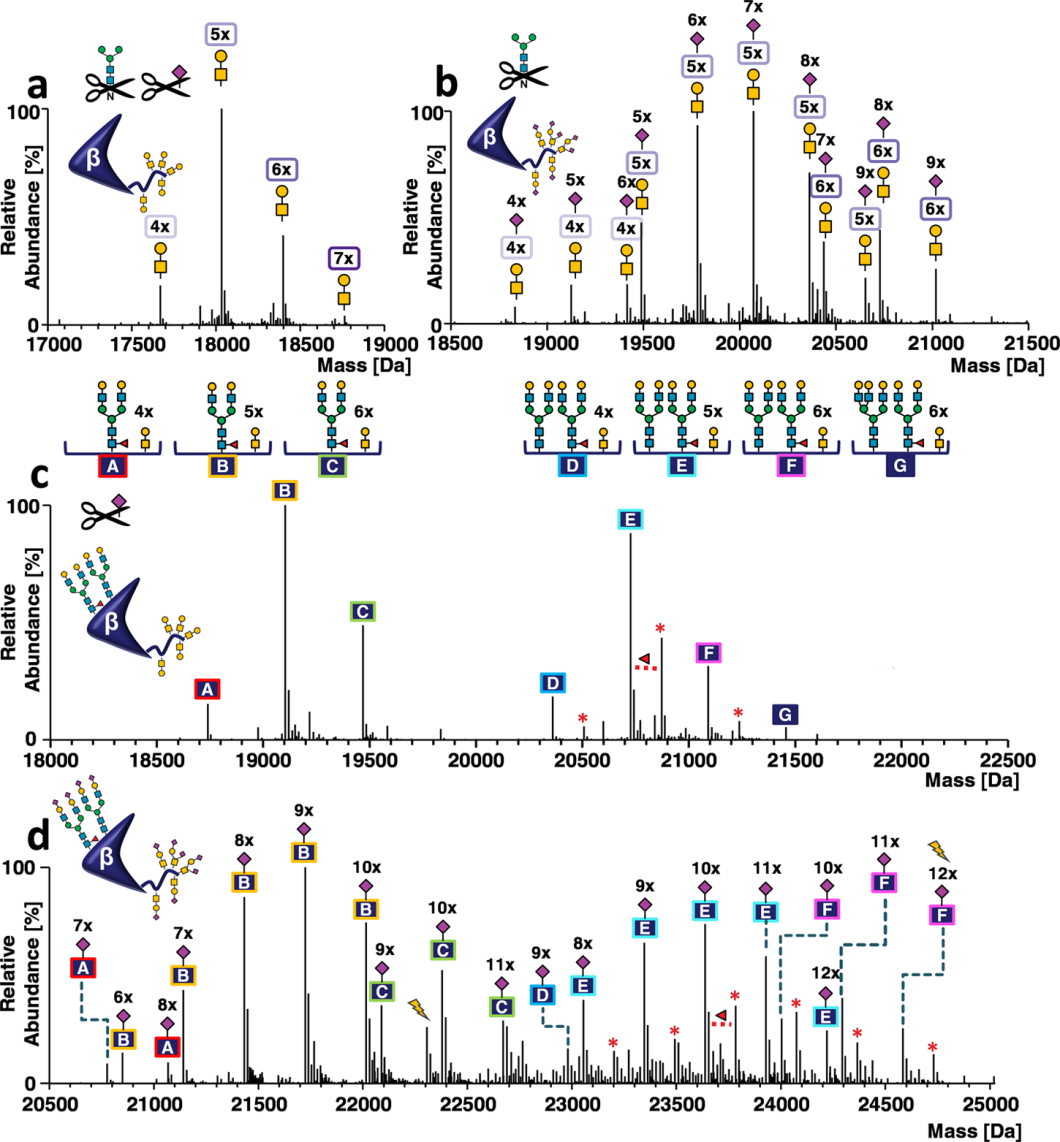
Annotation of hCGβ glycoforms. Deconvoluted mass spectra of carbamidomethylated hCGβ obtained by HPLC-MS analysis (a) after sialidase and PNGase F treatment, (b) after PNGase F treatment, (c) after sialidase treatment, and (d) in untreated form. Glycoforms are indicated by letters (A–F). Red asterisks indicate fucose variants. The number of Neu5Ac residues is indicated by the numbers above the peaks. Lightning bolts indicate two peaks that are further discussed in the main text. Peak lists with all possible glycoform assignments for untreated and desialylated hCGβ are available in the Supporting Information. Annotations: hCGβ BA059433. The instrument resolution setting was 140 000 at *m*/z 200.

For hCGβ, analysis of de-N-glycosylated and desialylated hCG revealed the number of O-glycan cores mainly ranging from four to six (Figure 3a). However, a glycoform carrying 7 O-glycans can also be observed. This finding is in disagreement with glycopeptide data (Figure S7), where two is the maximum number of O-glycans detected per peptide (maximum of six at the intact hCGβ level). In a next step, we analyzed PNGase F-treated hCG to obtain information on sialic acid variants of O-glycoforms (Figure 3b). Intriguingly, an hCGβ glycoform comprising five O-glycans and nine sialic acids was detected, which was not supported by glycopeptide data. This variant can only be explained when at least one O-glycopeptide carries two O-glycan cores with four sialic acids, but no such glycopeptide was detected (Figure S7). Subsequent analysis of sialidase-treated hCGβ revealed two abundant series of signals (Figure 3c). Using the approach described for the α subunit (in silico desialylated glycan library), we were able to assign desialylated N-glycoforms revealing the degree of galactosylation (Figure 3c). The two series of signals corresponded to glycoforms carrying one (letters A–C) or two N-glycans (letters D–G) and four to six O-glycan cores. Specifically, we detected biantennary N-glycan A2G2F (letters A–C, Figure 3c), the two biantennary N-glycans with one fucose A2G2/A2G2F (letters D–F, Figure 3c), or the two fucosylated biantennary N-glycans A2G2F/A2G2F (Figure 3c, glycoforms indicated by red asterisks). Glycopeptide data indicates a site occupancy of 62.6% at N13β and fucosylation of one-third of the N-glycans at this position. Moreover, site-specific differences were observed, as N30β glycans were almost completely fucosylated. These findings are also reflected in the intact spectrum of hCGβ. It is worth noting that the glycoform corresponding to letter G in Figure 3c, assigned as A2G2F/A3G3 + 6 core-1, can also be explained as A2G2F/A2G2 + 7 core-1 based on de-N-glycosylated and desialylated hCGβ (Figure 3a), but this assignment is not supported by glycopeptide data (Figure S7).

Finally, we analyzed sialylated N- and O-glycoforms of the intact hCGβ subunit. The deconvoluted mass spectrum of intact hCGβ (408 signals ranging from 20.5 to 25.0 kDa) exhibited a signal pattern that is explained by a combination of the identified N- and O-glycoforms, including terminal sialic acids, as revealed upon integration of the site-specific glycan library including sialic acids (letters A–F, Figure 3d). The complete annotation lists comprising 755 (hCGα) and 16 894 (hCGβ) entries can be found in Supporting Information, Annotations hCGα BA059433 and Annotations hCGβ BA059433.

Annotation of the deconvoluted hCGβ mass spectrum was successful and plausible for all but two peaks (lightning bolts in Figure 3d). The MoFi annotation of the peak at 22 306.2 Da was A3S2G1F + LacN1/M5-A1G1 and five core-1 with one sialic acid with a hit score of 100. From the PNGase F digest (Figure 3b), we know that no O-glycoform comprising five core-1 and only a single Neu5Ac exists. Considering that this peak shows a delta mass of +291 Da compared to the last peak of the B-series, it is likely that the correct annotation is unmodified/A2S2F and 5 core-1 + 9 S, which is supported by the PNGase F digest data. The wrong annotation stems from the absence of tryptic O-glycopeptides carrying two core-1 and four sialic acids in the site-specific glycan library, as this variant was not detected by nano-HPLC-MS/MS.

Additionally, no glycoform could initially be attributed to the mass of 24 583.7 Da using a mass tolerance of 20 ppm. Annotation was only possible when a mass tolerance of more than 44 ppm was set. The resulting glycoform A2S2/A2S2F and 6 core-1 + 8 S had a theoretical mass of 24 584.8 Da. This unusually high mass error was most likely caused due to an artifact of deconvolution resulting in a mass shift of −1 Da, a phenomenon often observed for proteins with a sulfur content above average.^48^

### Stepwise Data Integration of Structural Levels Facilitates Deciphering the Chemical Space of Dimeric hCG

By integrating the data of lower structural levels (released glycan, glycopeptide), we were able to elucidate and assign the glycosylation patterns of intact hCGα and hCGβ subunits. With this information in hand, we tackled the initially obtained native spectrum of the complex and investigated how the subunits combine within the heterodimer.

**Figure 4:**
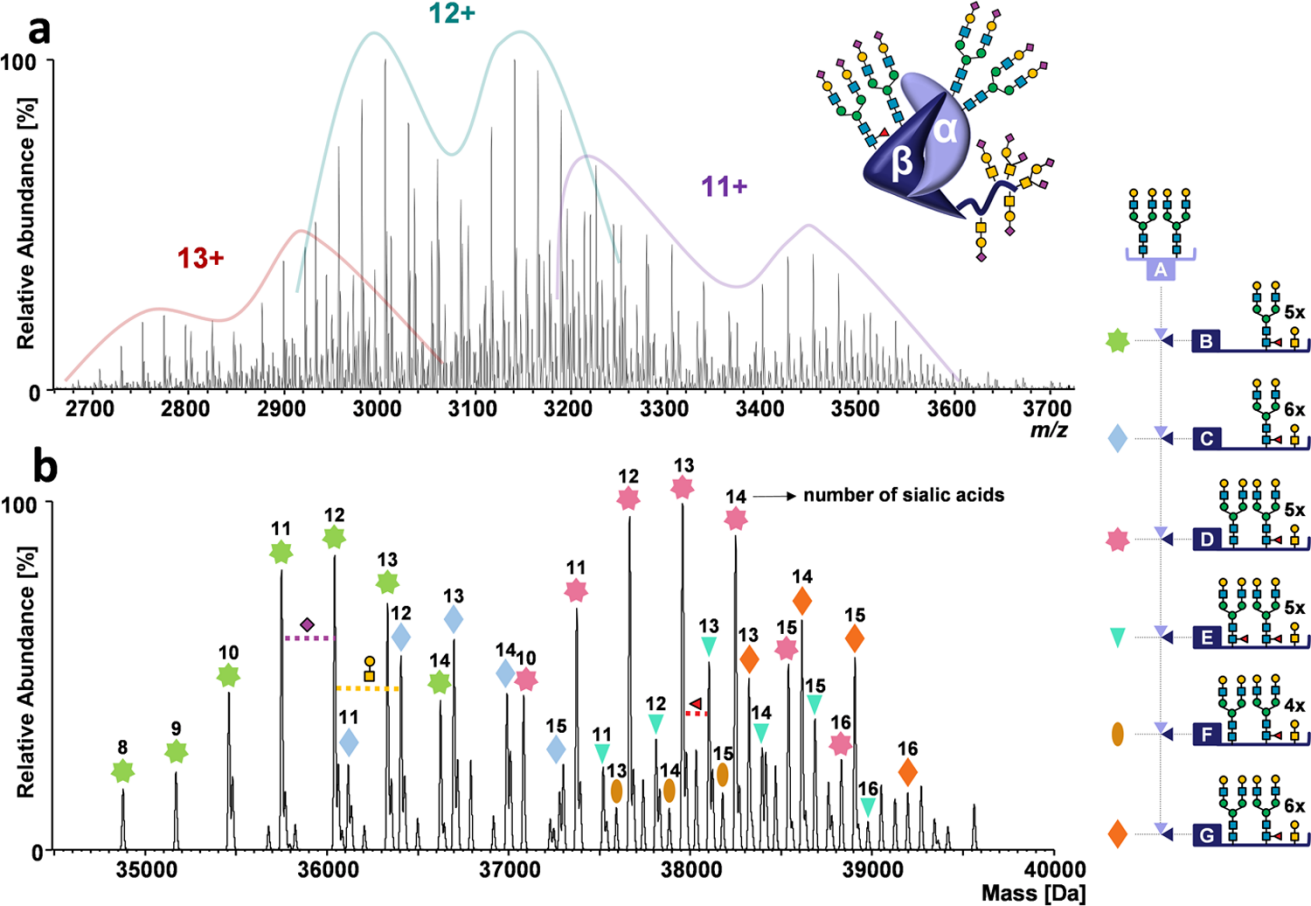
Annotation of dimeric hCG glycoforms. (a) Raw mass spectrum of dimeric hCG obtained by native MS. Charge states are indicated. (b) Deconvoluted mass spectrum of dimeric hCG. The glycoforms contributing most to peak abundance are indicated by colored symbols and represent a combination of glycans present on hCGα (A) and hCGβ (B–G). Numbers above these symbols correspond to the number of sialic acids present. The spectrum was obtained with an instrument resolution setting of *R* = 17 500 at *m*/*z* 200.

The raw spectrum (Figure 4a) showed three peak series in the *m*/*z* range between 2500 and 4000, corresponding to charge states from 11+ to 13+. The deconvoluted spectrum (Figure 4b) featured 48 signals in a mass range spanning from 34.5 to 39.6 kDa. Some peaks can be observed as doublets due to formation of sodium adducts (mass difference +22 Da), which may be attributed to incomplete buffer exchange. Analogous to the glycopeptide library, we built a library of hCGα and hCGβ glycoforms to be used in MoFi to annotate the deconvoluted mass spectrum of the hCG complex. By including more information on the molecular context of glycans, using subunit instead of glycopeptide data for the calculation of theoretically coexisting glycoforms of the hCG dimer, we were able to reduce the number of potential glycoforms by more than 17-fold (3 × 10^6^ instead of 51 × 10^6^ possibilities). In the case of hCG data, MoFi was limited to approximately 120 entries in the glycan list (since the combinatorial search space would explode with more entries). Thus, we decided to limit the number of glycoforms used for the library by employing a cutoff of 0.3% fractional abundance. Hence, the considered glycoforms (42 for hCGα and 58 for hCGβ) described 89.4 and 59.9% of the total glycoform abundance, respectively. For hCGβ, we assumed that the individual contributions of low-abundant subunit variants to peak intensity were negligible because 98.7% of the hCGβ glycoforms (10 984 out of 11 126) are each below the 0.1% fractional abundance threshold and make up only ≈35.7% of the total intensity. Thus, we assume that peak annotation was not significantly impacted by eradication of these glycoforms. When calculating the abundance of subunit glycoforms, we did not consider oxidation variants observed at intact level (Δ*m* = +16 Da) as distinct proteoforms and summed the abundances of the corresponding non-oxidized and oxidized species. Using the subunit library, 1031 glycoforms were identified for 48 detected masses (a complete list of annotations can be found in Supporting Information, Annotations Dimer BA059433).

In the deconvoluted spectrum of dimeric hCG shown in Figure 4b, the variant explaining the highest portion of the abundance of each peak (as calculated by MoFi) is reported. Most N-glycoforms were composed of sialylated biantennary complex type glycans, while triantennary N-glycans were observed to a lesser extent. As expected from subunit data, the deconvoluted spectrum shows five main signal series of glycovariants with delta masses of 291 Da, corresponding to a varying number of sialic acid residues. The two main patterns (green and pink stars in Figure 4b) are due to sialic acid variants of N-glycans with biantennary structures. A delta mass of 365 Da is also observable and may be attributed to variants with an additional antenna (GlcNAc_1_Gal_1_ for N-glycans or GalNAc_1_Gal_1_ for O-glycans; light blue and orange rhomboids in Figure 4b).

Moreover, a difference in mass of 146 Da was observed arising from variants comprising two fucosylated N-glycans on hCGβ (turquoise triangles and brown ovals in Figure 4b). At an instrument resolution setting of 17.500, it is not possible to distinguish between a glycoform comprising a sialic acid (291 Da) or two fucose (292 Da) residues based on the delta mass. Since all abundant variants carried sialic acid residues, enzymatic desialylation was performed in order to clarify these mass differences. After desialylation, the raw spectrum (Figure S11a) showed significantly lower complexity compared to the intact dimer. Charge state distribution was practically unaffected by removal of sialic acids, as the three main charge states remained at 11+ to 13+. The deconvoluted mass spectrum (Figure S11b) showed 13 peaks in a mass range between 31 and 36 kDa. In Figure S11b, the most abundant hCG glycoforms are reported as a combination of hCGα and β glycovariants (the complete annotation is available in Supporting Information, Annotations Dimer BA059433). No mass differences of 291 or 292 Da were observed in this spectrum, confirming the mass shifts to arise from sialic acids and not multiple fucoses in the spectrum of the untreated complex (e.g. pink stars in Figure 4b).

### Glycoform Annotations Are Relevant for Biopharmaceutical Quality Control

In-depth hCG characterization of the second Ovitrelle batch (Supporting Information, Annotations Dimer, hCGα or hCGβ, BA056714) allowed the detailed assessment of glycosylation for batch-to-batch comparison at multiple structural levels. First, we decided to derive rather global parameters such as the overall degree of sialylation and core fucosylation from the glycoform annotation results. To obtain the degree of sialylation, we divided the amount of sialic acids observed in a given glycopeptide or glycoform by the calculated maximum number of sialic acids that could be present on this glycopeptide/glycoform (one per N-glycan antenna, two per O-glycan). All ratios were weighted for abundance to calculate the mean degree of sialylation. Core fucosylation was addressed in a similar fashion by considering a maximum of one core fucose per N-glycan. Indeed, core-fucosylation and sialylation degrees were consistent between the two Ovitrelle batches across all structural levels (see Table 2).

**Table 2:** Comparison of the Degrees of Core Fucosylation and Sialylation in Both Ovitrelle Batches at Different Structural Levels

		degree of core fucosylation		degree of sialylation	
structural level	target	BA059433	BA056714	BA059433	BA056714
glycopeptides	hCGα	0.028	0.026	0.61	0.59
	hCGβ	0.66	0.68	0.63	0.63
subunits	hCGα	0.10	0.71	0.65	0.64
	hCGβ	0.71	0.73	0.67	0.67
dimer	hCG dimer	0.33	0.34	0.71	0.71

Looking at individual glycoform signals, slight apparent differences in the abundance of glycoforms were observable in the deconvoluted native spectra of Ovitrelle batches (Figure 1) and were verified by comparing the charge state signals in the raw spectra (Figure S1). Batch BA059433 showed a higher abundance of glycoforms lacking N-glycosylation at N13β (signal series ranging from 34.8 to 37.1 kDa in Figure 1). In contrast, fully N-glycosylated glycoforms were more prominent in BA056714 (signal series ranging from 37.1 to 39.6 kDa in Figure 1). However, after calculating the fractional abundance of glycoforms according to MoFi score and relative peak abundance, we observed that the most abundant peak (37967.0 Da for BA059433 and 36052.6 Da for BA056714) did not contain the most abundant glycoform. Interestingly, the variant A2S1G1/A2S2/unmodified/A2S2F/1 × Core + 2 × S/2 × Core + 3 × S/2 x Core + 3 × S with a mass of 36 343.5 Da (Figure 1) was identified as the most abundant for both batches, despite the relative abundance of the corresponding mass peak in the deconvoluted spectrum of the hCG dimer being 71.1% for BA059433 and 73.0% for BA056714.

## Conclusions

The integration of comprehensive data on protein glycosylation from different structural levels facilitates the exploration of the chemical space of highly heterogeneous glycoforms in protein complexes and provides valuable insights into the structural intricacies of glycoproteins. Analyzing intact hCG subunits and the hCG dimer highlights the advantage of intact protein/protein complex analysis over glycopeptide analysis, because the structural context of microheterogeneity as well as macroheterogeneity remains preserved and allows for direct assessment of specific glycoforms. Moreover, in analyzing the noncovalent protein complex of hCG consisting of highly glycosylated subunits, we took advantage of two assets of native MS—the increased spatial resolution and the preservation of noncovalent interactions. The resulting mass spectrum discloses the heterogeneity of the hCG heterodimer and provides detailed information on glycoforms of the intact complex.

Although the number of distinguishable signals in the mass spectrum of the intact complex is in the range of only 50, computer-aided integration of the semiquantitative data of released glycans, glycopeptides, and protein subunits facilitates the assignment of more than 1000 underlying glycoforms that can explain the experimentally observed pattern of proteoforms. The discrepancy between the number of observed signals and the number of underlying proteoforms rests within (1) the fundamental inability of MS to resolve isobaric proteoforms as well as (2) overlapping isotope clusters of variants having small mass differences. This prevents the distinction of proteoforms having mass differences in the order of 25 Da or less for a 38 kDa protein, irrespective of the achievable mass spectrometric resolution (see ref (16)).

Nevertheless, our probability-based calculation of the contribution of distinct glycoforms to the overall observed signal intensity (hit scores in Supporting Information, Annotations Dimer BA059433) reveals that in most cases, a limited number of five different glycoforms contribute to the major portion of the observed intensity in a distinct signal. Hence, about 385 different glycoforms of more than 0.04% fractional abundance explain 90% of all signal intensities in the glycoform pattern shown in Figure 4b.

Finally, we successfully demonstrate the capability of our approach to investigate drug product quality attributes. The obtained glycoprotein profiles may serve as fingerprints, e.g. in the assessment of batch-to-batch variability. We believe that advancement in instrument and software performances will increase the robustness of the approaches involving native MS and enable quick characterization of glycosylated biopharmaceuticals at the intact level in the future.

## Supporting Information Available

Mirror plot of native raw mass spectra of dimeric hCG of two batches of Ovitrelle; workflow used to characterize hCG; electropherogram of released N-glycans; extracted ion chromatogram of O-glycans after chemical release; results of HPLC-MS/MS analysis of two Ovitrelle batches upon tryptic digestion; total ion current chromatogram of Ovitrelle analysed by RP-HPLC-MS; raw mass spectra of intact and enzymatically dissected hCG subunits obtained by HPLC-MS analysis and annotation of dimeric hCG glycoforms after removal of sialic acid. (PDF)
Glycan names, structure, mass and electropherogram peak number of Ovitrelle batches BA059433 and BA056714 (XLSX)
Results of Byonic for glycopeptide identification Ovitrelle batches BA059433 and BA056714 (ZIP)
Results of MoFi annotations for subunits and dimer, Ovitrelle batches BA059433 and BA056714 (ZIP)

## Notes

Raw files and MoFi settings are available from Zenodo (DOI: 10.5281/zenodo.4320966). All input files and data analysis scripts used in this study are freely available from GitHub (https://github.com/cdl-biosimilars/hcg-glycosylation).
