# Supplementary material for: Exploring the Chemical Space of Protein Glycosylation in Noncovalent Protein Complexes: An Expedition along Different Structural Levels of Human Chorionic Gonadotropin by Employing Mass Spectrometry
Source: Anal Chem. 2021 Jul 21;93(30):10424–34. doi: 10.1021/acs.analchem.1c02199 (PMC8340079; doi:10.1021/acs.analchem.1c02199)
Supplement: Supplementary file 1 — ac1c02199_si_001.pdf [file ac1c02199_si_001.pdf]

Supporting Information

Exploring the Chemical Space of Protein Glycosylation in Noncovalent Protein Complexes: An Expedition along Different Structural Levels of Human Chorionic Gonadotropin by Employing Mass Spectrometry

Maximilian Lebede,[||1,2] Fiammetta Di Marco,[||1,2] Wolfgang Esser-Skala,[1,2,3] René Hennig,[4,5] Therese Wohlschlager,[1,2] Christian G. Huber*[1,2]

[1]Department of Biosciences, Bioanalytical Research Labs, University of Salzburg, Hellbrunner Straße 34, 5020 Salzburg, Austria

[2]Christian Doppler Laboratory for Innovative Tools for Biosimilar Characterization, University of Salzburg, Hellbrunner Straße 34, 5020 Salzburg, Austria

[3]Department of Biosciences, Computational Systems Biology Group, University of Salzburg, Hellbrunner Straße 34, 5020 Salzburg, Austria

[4]glyXera GmbH, Brenneckestraße 20 - ZENIT, 39120 Magdeburg, Germany

[5]Max Planck Institute for Dynamics of Complex Technical Systems, Sandtorstraße 1, 39106 Magdeburg, Germany

|| These authors contributed equally.

* Email: c.huber@sbg.ac.at

Table of Contents for Supporting Information

Figure S1 .... S3
Figure S2 .... S4
Figure S3 .... S5
Figure S4 .... S6
Figure S5 .... S7
Figure S6 .... S8
Figure S7 .... S9
Figure S8 .... S10
Figure S9 .... S11
Figure S10 .... S12
Figure S11 .... S13

SUPPLEMENTARY FIGURES

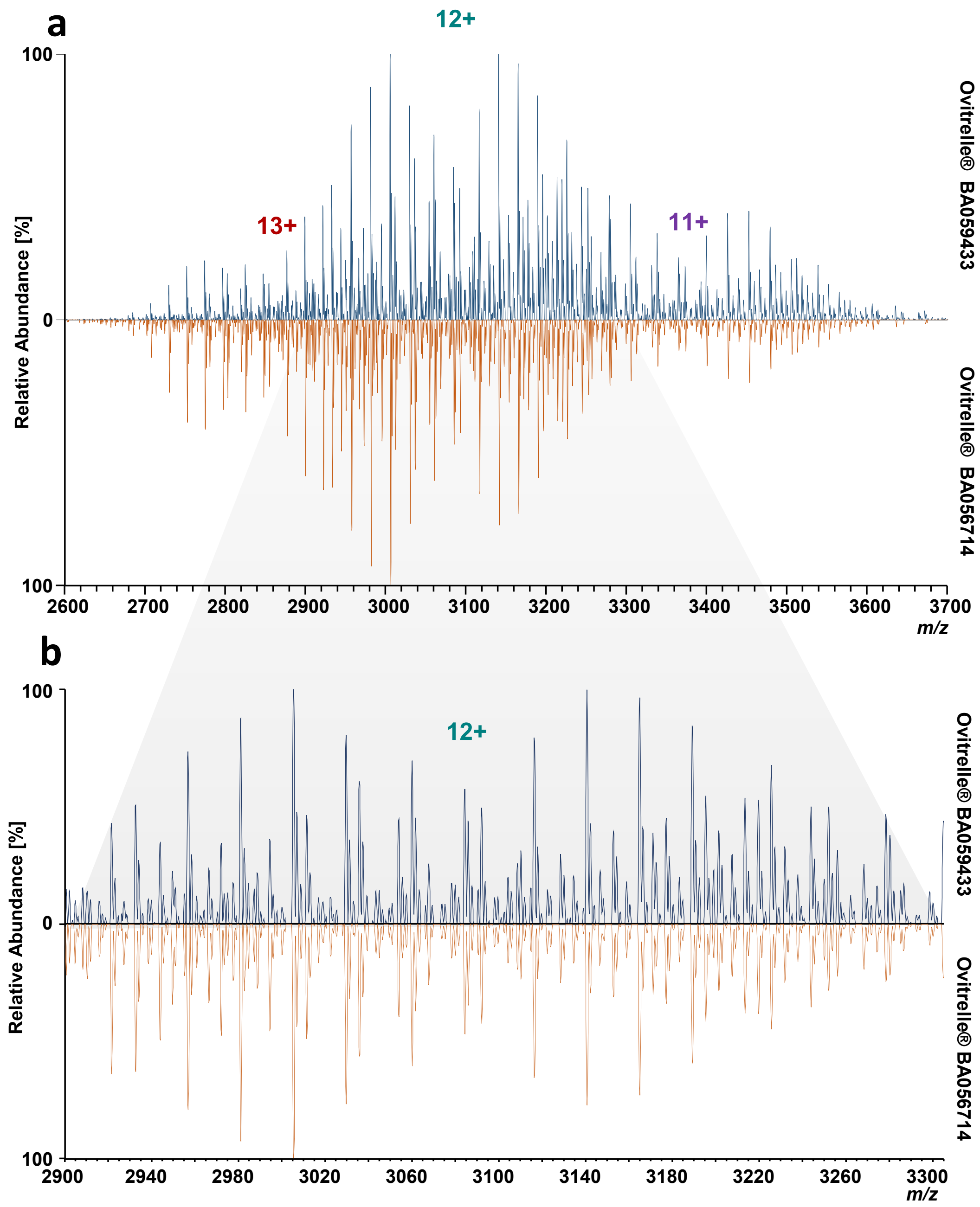

Figure S1 Mirror plot of native raw mass spectra of dimeric hCG of two batches of Ovitrelle (BA059433, BA056714). **a** Raw mass spectra of the two Ovitrelle batches. Three main charge states are present, *i.e.* 11+ to 13+. **b** Magnification of the most abundant charge state 12+. Differences of relative peak intensities can be observed between the two batches (see Figure 1 in the main manuscript for deconvoluted mass spectra). Spectra were obtained with an instrument resolution setting of R=17,500 at 200 *m/z*.

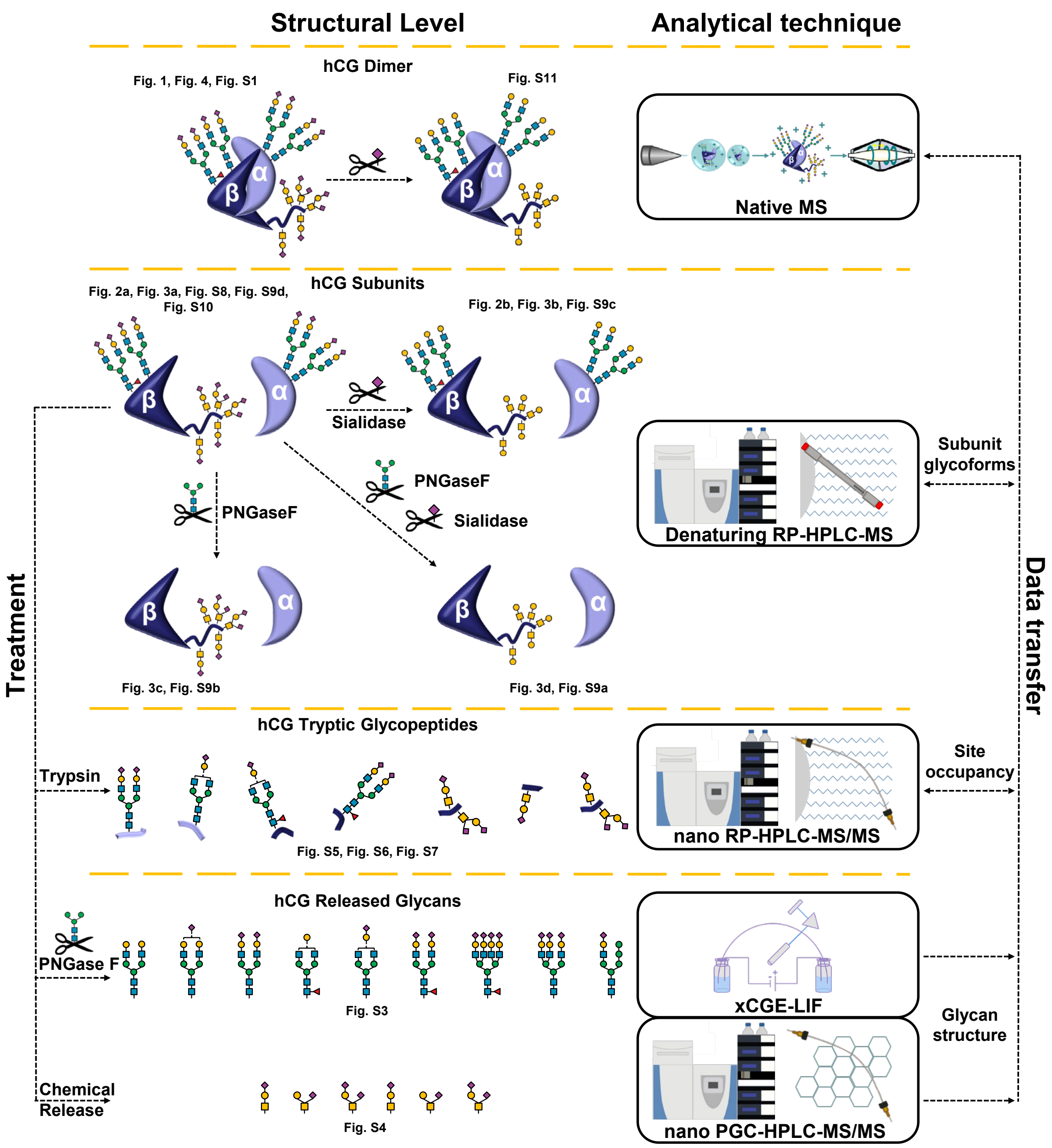

Figure S2 Representation of the workflow used to characterize hCG at different structural levels and upon enzymatic dissection. Analytical techniques used are indicated on the right side. Figure numbers of associated electropherograms, chromatograms and mass spectra are reported for every structural level.

PGC-HPLC-MS/MS, porous graphitized carbon high-performance liquid chromatography-tandem mass spectrometry

xCGE-LIF, multiplexed capillary gel electrophoresis-laser-induced fluorescence

RP-HPLC-MS/MS, reversed-phase high-performance liquid chromatography-tandem mass spectrometry

RP-HPLC-MS, reversed-phase high-performance liquid chromatography-mass spectrometry

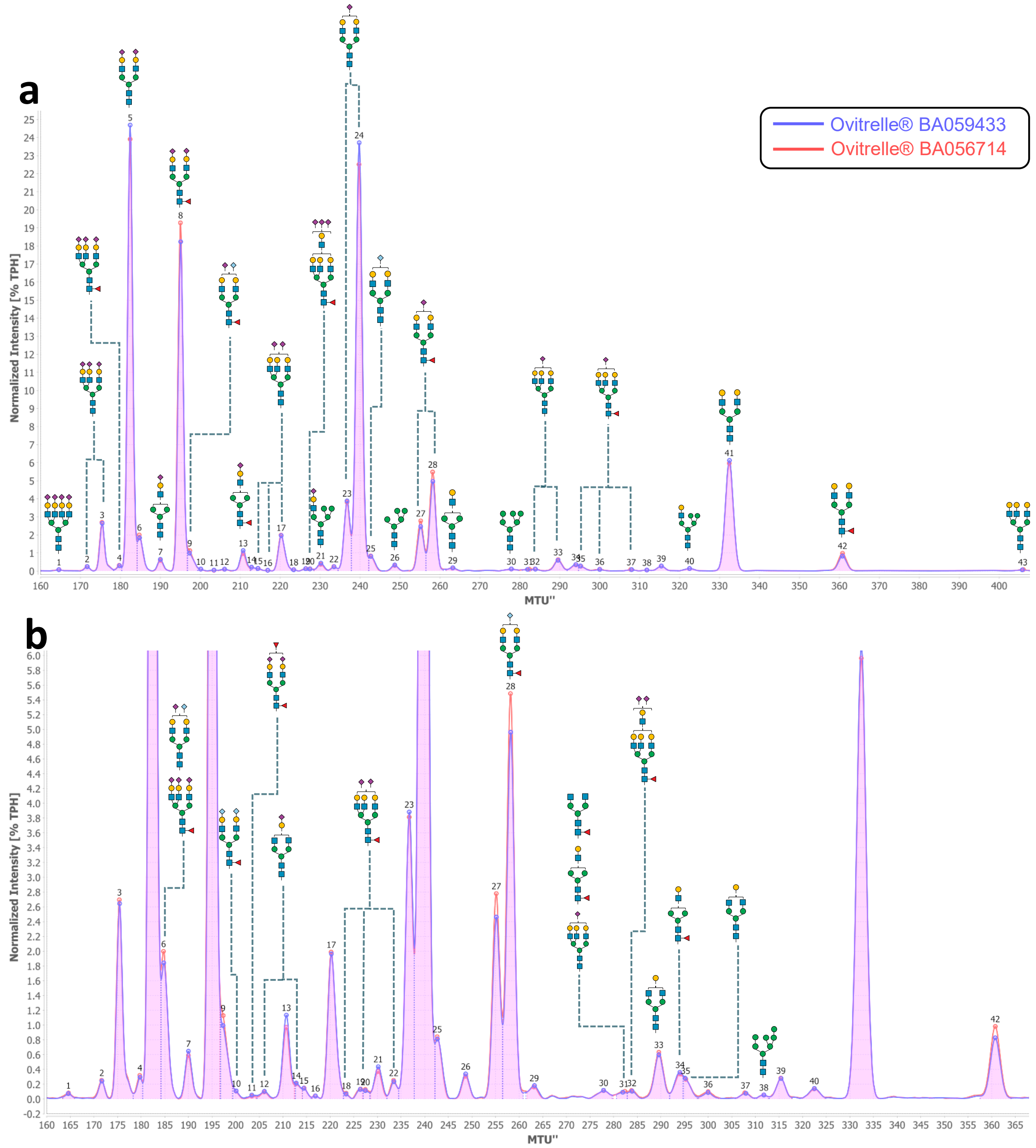

Figure S3 **a** Electropherogram obtained for released N-glycans of Ovitrelle Batches BA059433 (blue) and BA056714 (red) by multiplexed capillary gel electrophoresis with laser induced fluorescence detection (xCGE-LIF). Peaks are numbered from 1–43 and the corresponding identified N-glycans are reported. **b** Magnification of the low abundant peaks. Glycan structures, names and compositions are collected in Supporting Information, Released glycan data.

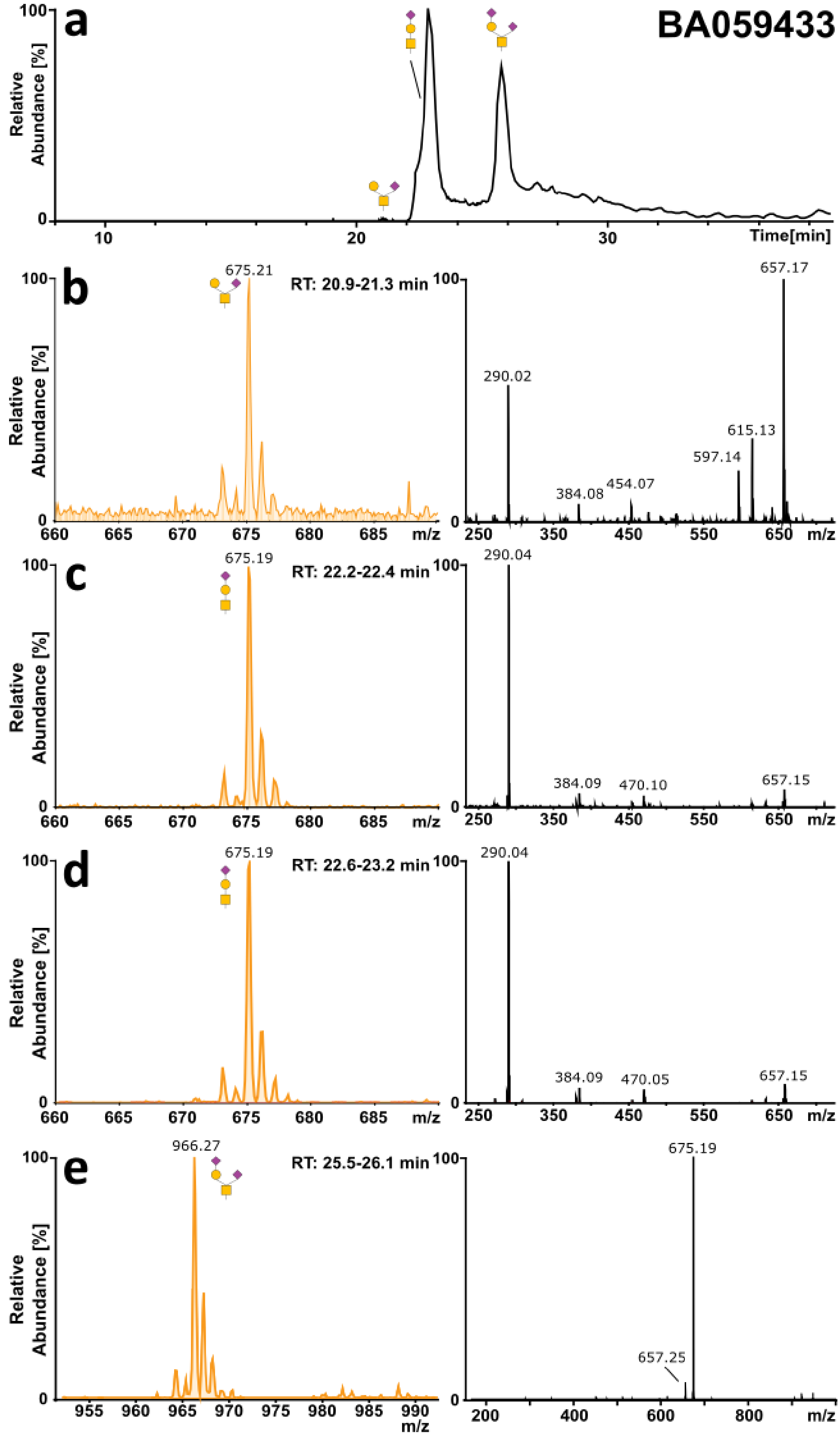

Figure S4 **a** Extracted ion chromatogram of O-glycans after chemical released analyzed by nano-PGC-HPLC-MS/MS. Identified structures are shown. **b-e** Appendant mass spectra of O-glycans at different retention times (left side) and corresponding MS2 fragment spectra (right side). Glycan structures, names and compositions are collected in Supporting Information, Released glycan data.

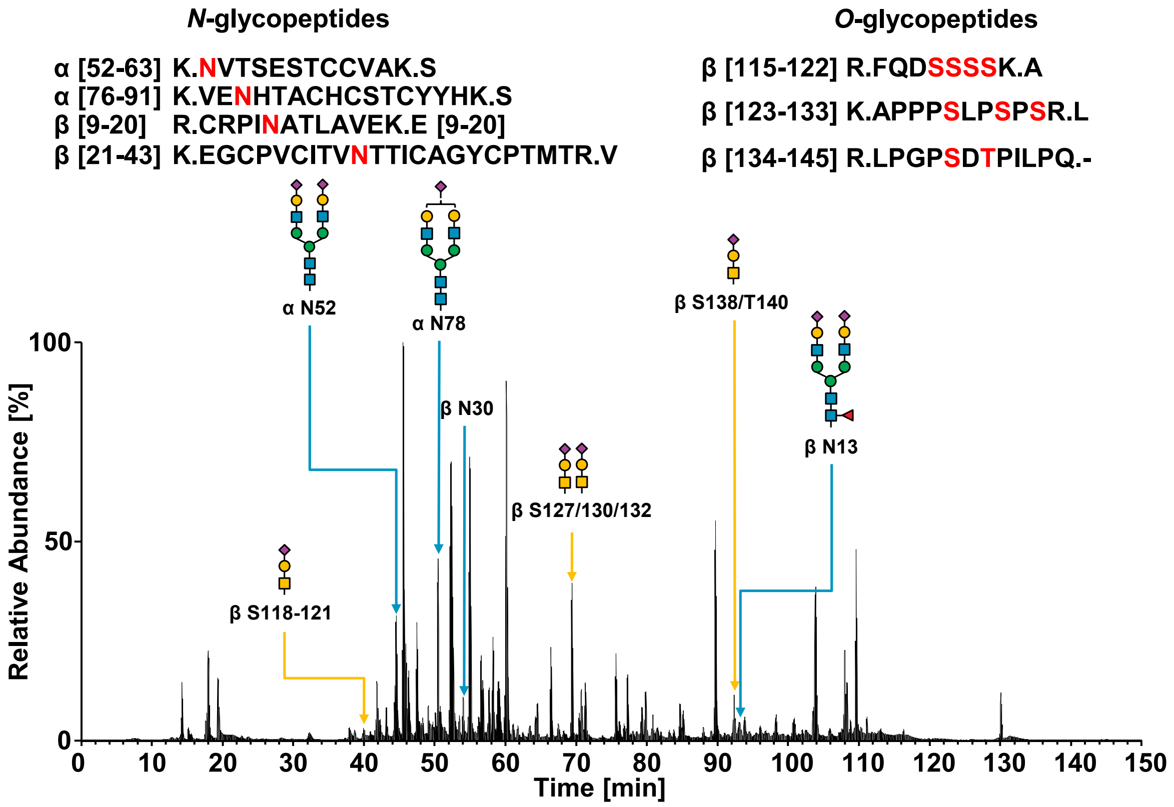

Figure S5 Total ion current chromatogram of Ovitrelle® Batch BA059433 obtained by nano-RP-HPLC-MS/MS analysis upon tryptic digestion. Sequence of glycopeptides are shown and tryptic cleavage sites by dots. Corresponding subunits are indicated with α/β and glycosylation sites are specified by red letters. O-glycosylation site identification was ambiguous due to multiple O-glycosylation sites in the same peptide. The most abundant glycan for every peptide and corresponding peaks are indicated.

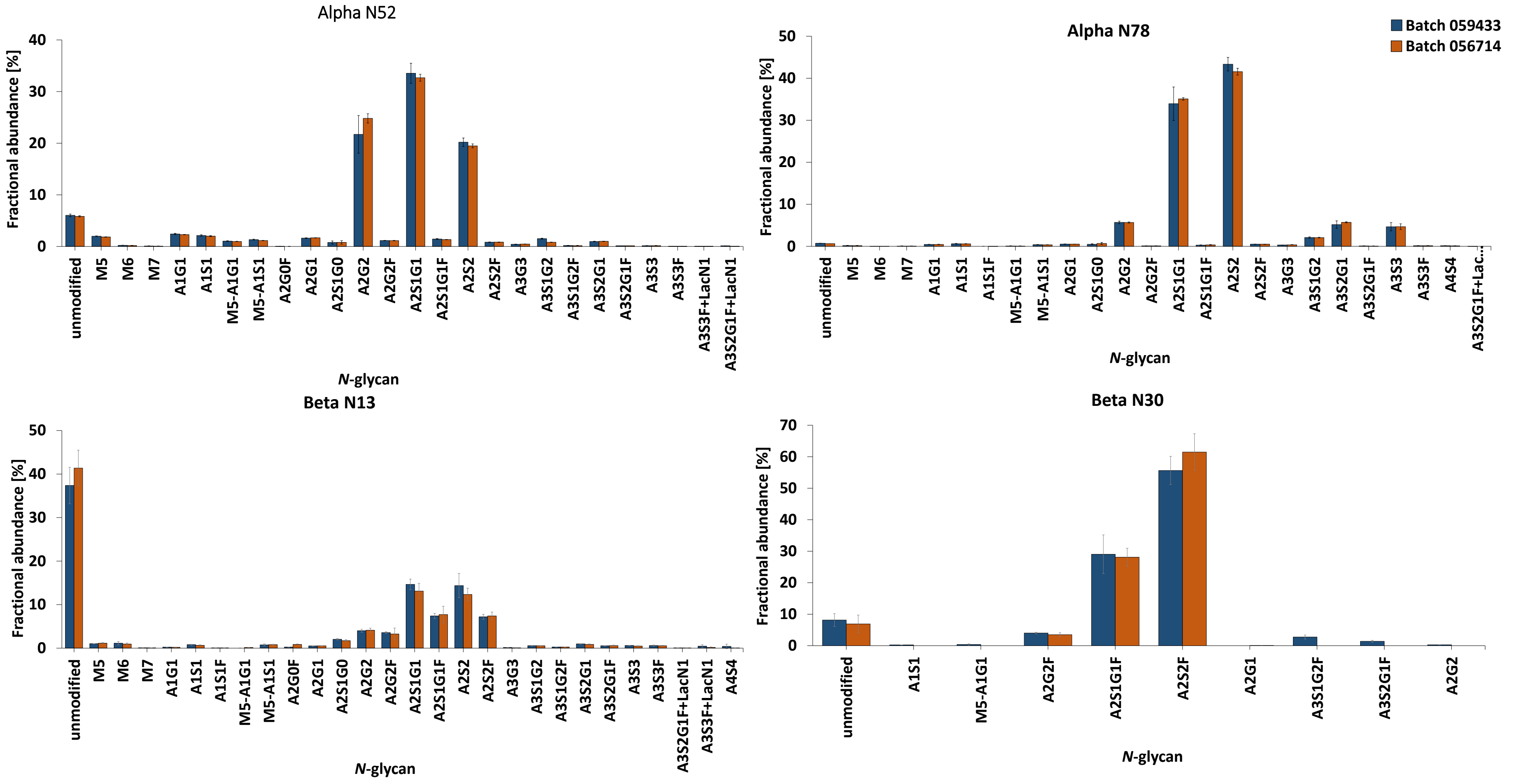

Figure S6 Results of HPLC-MS/MS analysis of two Ovitrelle® batches upon tryptic digestion. Bar charts depict fractional abundances of different glycan structures for each glycosylation site of hCGα (N52, N78) and hCGβ (N13, N30). Regarding hCGα, N52 and N78 exhibited a higher abundance of biantennary hybrid N-glycans with a different degree of sialylation between the two sites. While N52 comprised monoantennary and high mannose N-glycan structures, N78 exhibited a higher abundance of triantennary glycans. Regarding hCGβ N-glycans, modification at N13 occurred only at a relative abundance of ≈60% and is mostly modified by biantennary N-glycans (mono- and disialylated). Core fucosylation occurred at these glycans with a relative abundance of ≈50% compared to the unfucosylated counterparts. N30β showed less complexity compared to the other sites, carrying mainly core fucosylated biantennary N-glycans. No clear differences were observed between the two batches. Glycan structures, names and compositions are collected in Supporting Information, Released glycan data.

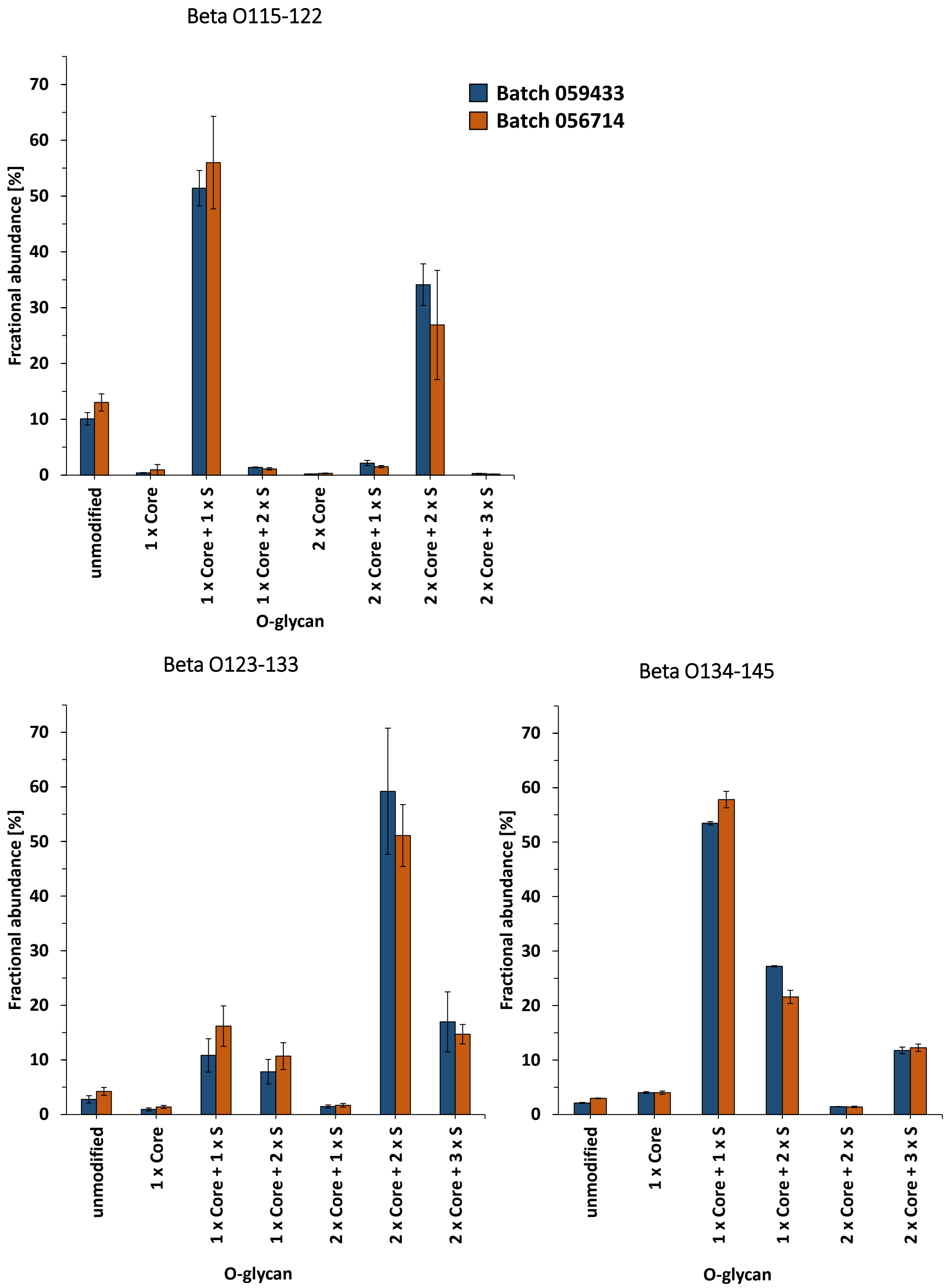

Figure S7 Results of HPLC-MS/MS analysis of two Ovitrelle batches upon tryptic digestion. Bar charts report fractional abundances of O-glycan structures of hCG*β* peptides (O115-122, O123-133, O134-145). Data for the three peptides of the hCG*β* C-terminus are reported. Every of the mentioned peptides carried more than one O-glycosylation sites (4, 3 and 2, respectively). The peptide O115-122 showed glycosylation of one or two O-glycosylation sites (≈50% and ≈30% relative abundance), meaning that all four serines cannot be simultaneously modified within a single glycoform. The same assumption is made for glycopetide O123-133, where two to three sites are simultaneously modified. Glycopeptides O123-133 and O134-145 showed a higher degree of sialylation compared to peptide O115-123. No clear differences were observed between the two batches. Glycan structures, names and compositions are collected in Supporting Information, Released glycan data.

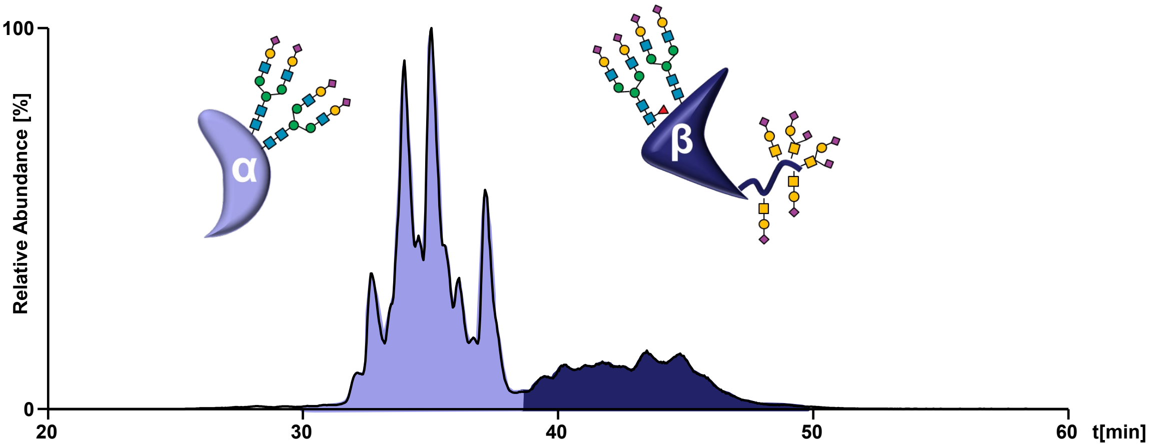

Figure S8 Total ion current chromatogram obtained by RP-HPLC-MS analysis of Ovitrelle. Peaks corresponding to hCG*α* are colored in light blue, peaks corresponding to hCG*β* are colored in dark violet. See Figure S9d for the corresponding spectra.

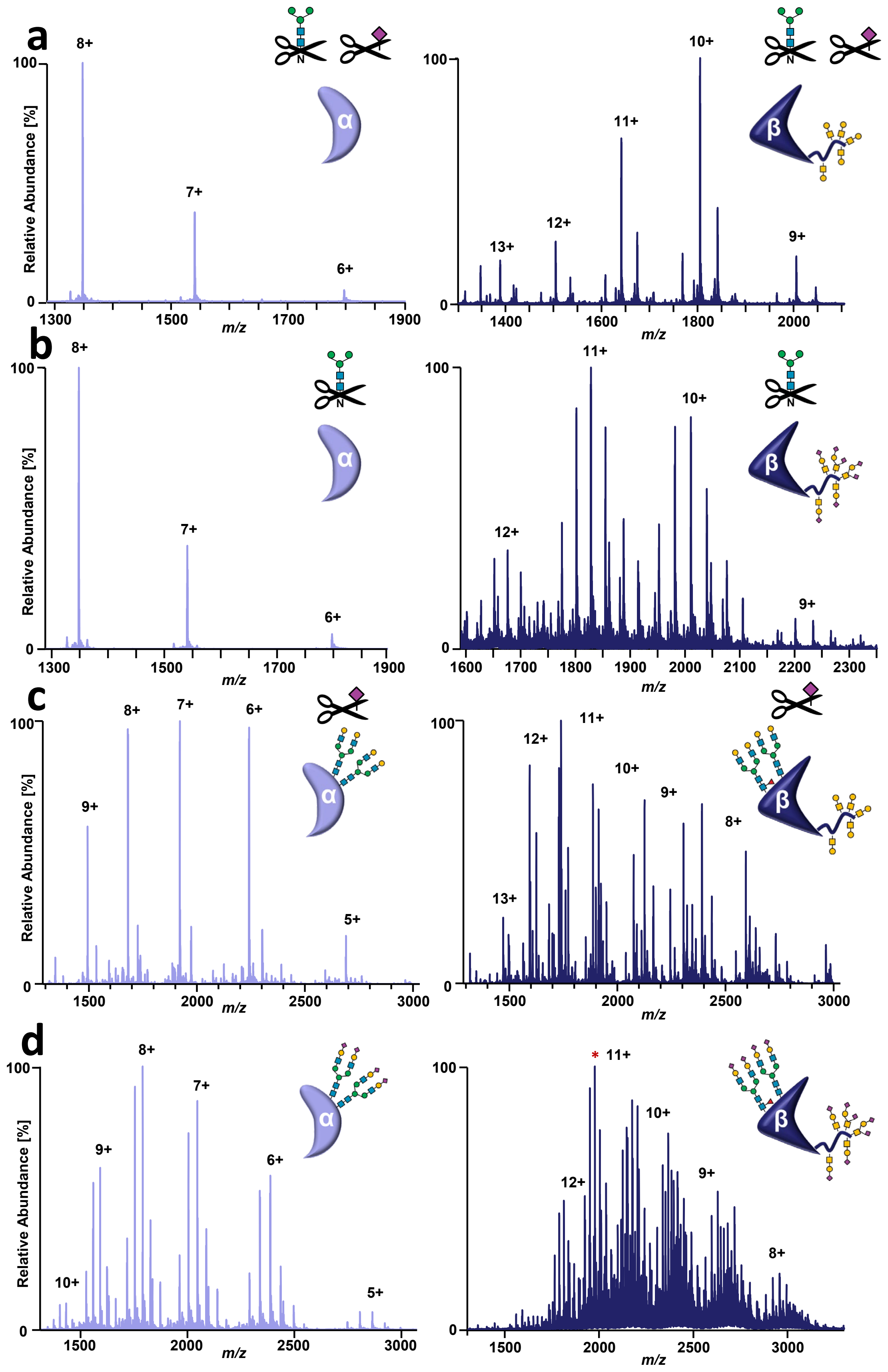

Figure S9 Raw mass spectra of intact and enzymatically dissected hCG subunits obtained by HPLC-MS analysis. Mass spectra of hCG*α* and hCG*β* are indicated in light blue and dark violet, respectively. **a** Raw spectra of reduced and carbamidomethylated hCG*α* and hCG*β* after treatment with PNGase F and sialidase. Multiple hCG*β* signals per charge state are due to the presence of desialylated O-glycans. **b** Raw spectra of reduced and carbamidomethylated hCG*α* and hCG*β* spectra after removal of N-glycans. Multiple hCG*β* signals per charge state are due to the presence of O-glycans. **c** Raw spectra of desialylated hCG*α* and hCG*β*. **d** Raw spectra of intact hCG*α* and hCG*β*. The red asterisk indicates the most abundant signal in charge state 11+ magnified in Figure S10. Spectra were obtained with an instrument resolution setting of R=140,000 at *m/z* 200.

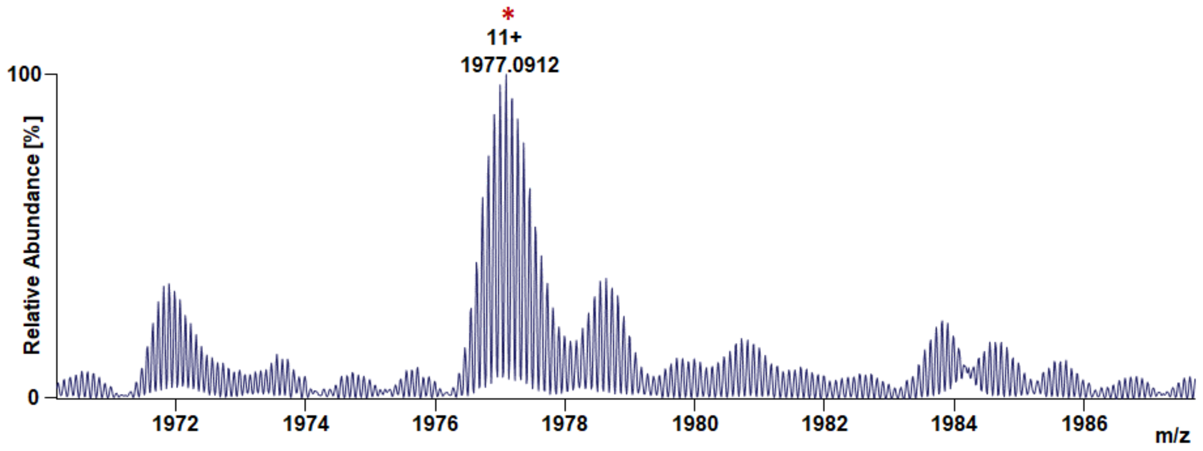

Figure S10 Magnification of the most abundant charge state of the raw mass spectrum of intact hCGβ (see red asterisk in Figure S9d, right side). The resolved isotopic pattern can be observed. Spectrum was obtained with an instrument resolution setting of R=140,000 at 200 *m/z*.

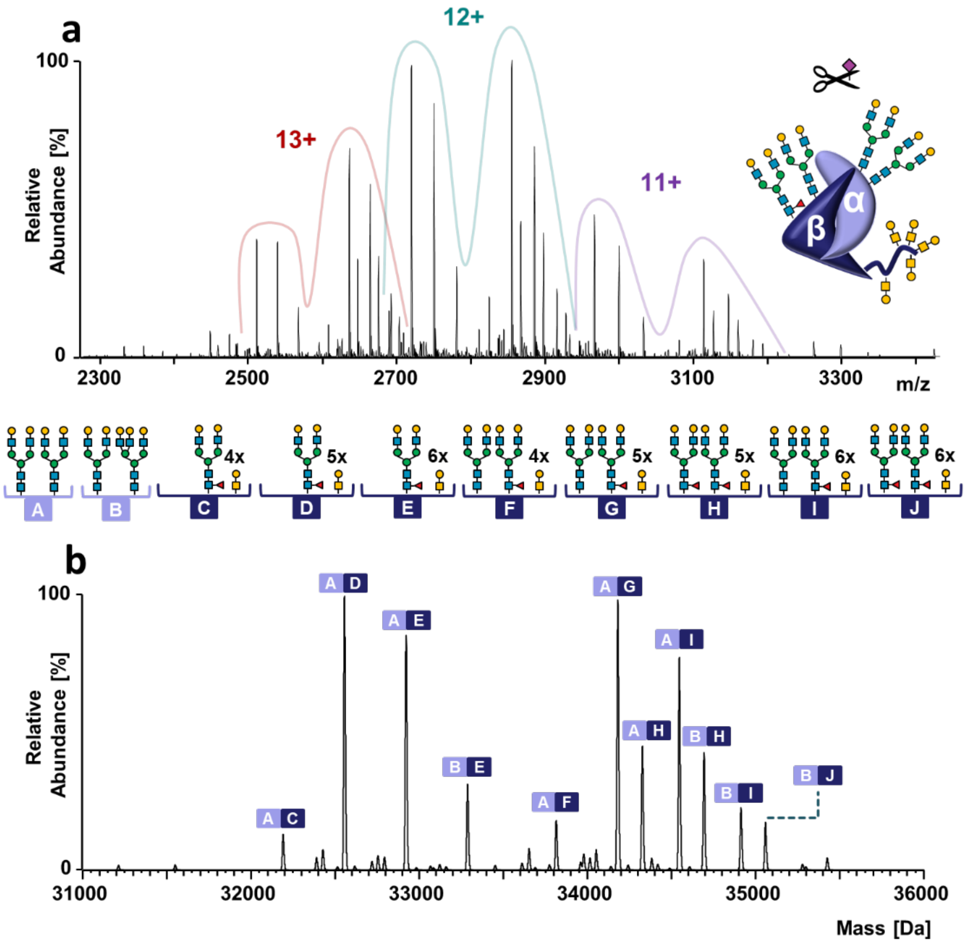

Figure S11 Annotation of dimeric hCG glycoforms after removal of sialic acid using sialidase. **a** Raw mass spectrum of sialidase-treated dimeric hCG obtained by native MS. Charge states are indicated. **b** Deconvoluted mass spectrum of sialidase-treated dimeric hCG. The glycoforms contributing most to peak abundances are indicated by colored squares and represent a combination of glycans present on hCG*α* (A–B) and hCG*β* (C–M). Spectra were obtained with an instrument resolution setting of R=17,500 at 200 *m/z*.
